# The hidden threats posed by Japanese encephalitis virus genotype V

**DOI:** 10.1128/jvi.01644-25

**Published:** 2025-11-04

**Authors:** Qi Li, Hridesh Mishra, Kevin C. Kain, Ran Wang

**Affiliations:** 1 Laboratory of Infection and Virology, Beijing Pediatric Research Institute, Beijing Children’s Hospital, Capital Medical University, National Center for Children’s Health 12517, Beijing, China; 2 Research Unit of Critical Infection in Children, 2019RU016, Chinese Academy of Medical Sciences, Beijing, China; 3 Beijing Key Laboratory of Core Technologies for the Prevention and Treatment of Emerging Infectious Diseases in Children, Beijing, China; 4 Department of Endocrinology and Metabolism, The First Hospital of China Medical University, Shenyang, Liaoning, China; 5 Sandra A. Rotman (SAR) Laboratories, Sandra Rotman Centre for Global Health, University Health Network-Toronto General Hospital 7989, Toronto, Ontario, Canada; 6 Tropical Disease Unit, Division of Infectious Diseases, Department of Medicine, University of Toronto 7938, Toronto, Ontario, Canada; 7 Department of Experimental Therapeutics, University Health Network-Toronto General Hospital 7989, Toronto, Ontario, Canada; 8 Faculty of Medicine, University of Toronto 12366, Toronto, Ontario, Canada; Indiana University Bloomington, Bloomington, Indiana, USA

**Keywords:** Japanese encephalitis virus, genotype V, phylogenetic analysis, pathogenicity, vaccine

## Abstract

Japanese encephalitis virus (JEV) remains a major cause of viral encephalitis in Asia. Among its five genotypes (GI–GV), genotype V (GV) has re-emerged and become the predominant lineage in mosquitoes in the Republic of Korea, with sporadic human cases also reported. GV JEV displays differences from other genotypes in genomic sequence, antigenicity, and pathogenicity, and animal studies indicate higher lethality. Importantly, all currently used vaccines are based on GIII strains and provide partial but insufficient protection against GV JEV, with breakthrough infections documented. These findings raise concerns that GV circulation could undermine existing control strategies. Although shifts in mosquito vectors have been observed, the broader epidemiological and clinical impact of GV JEV remains poorly defined. In the absence of specific antiviral therapies, systematic evaluation is needed to determine whether GV-based vaccines are warranted. This review summarizes current evidence on the epidemiology, molecular and biological characteristics, and vaccine protection of GV JEV, and highlights priorities for surveillance and future research.

## INTRODUCTION

Japanese encephalitis virus (JEV), a member of the *Flaviviridae* family and *Orthoflavivirus* genus, is one of the leading causes of viral encephalitis in Asia. According to the World Health Organization (WHO), JEV circulates in 24 countries across the WHO South-East Asia Region and Western Pacific Region, causing approximately 20,000 infections annually and placing over 3 billion people at risk of infection (1, 2). The majority of infected individuals are asymptomatic, while a small proportion develop Japanese encephalitis (JE), a severe neurological disease with a fatality rate of ~30%. Moreover, ~50% of JE survivors experience permanent neurological, cognitive, and behavioral sequelae. However, no specific antiviral treatments are currently available for JE (3). Fortunately, JE is a vaccine-preventable disease, and the widespread promotion and accessibility of vaccination programs have significantly reduced the incidence of JE globally over the past two decades (4).

JEV can be classified into five genotypes: genotype I (GI), GII, GIII, GIV, and GV (5). Prior to the 1980s, GIII JEV was the predominant genotype circulating in Asia and was associated with multiple JE epidemics. However, throughout the 1980s into the 2000s, GI JEV gradually replaced GIII JEV as the dominant genotype, causing JE outbreaks in East Asia and Southeast Asia (6, 7). GII JEV has rarely caused outbreaks or localized transmission, with sporadic cases reported in Indonesia, the Republic of Korea (ROK), Malaysia, and Australia (8). In 2022, GIV, which has long been overlooked, re-emerged in Australia, leading to localized JE outbreaks and several fatal cases (9, 10).

**Fig 1:**
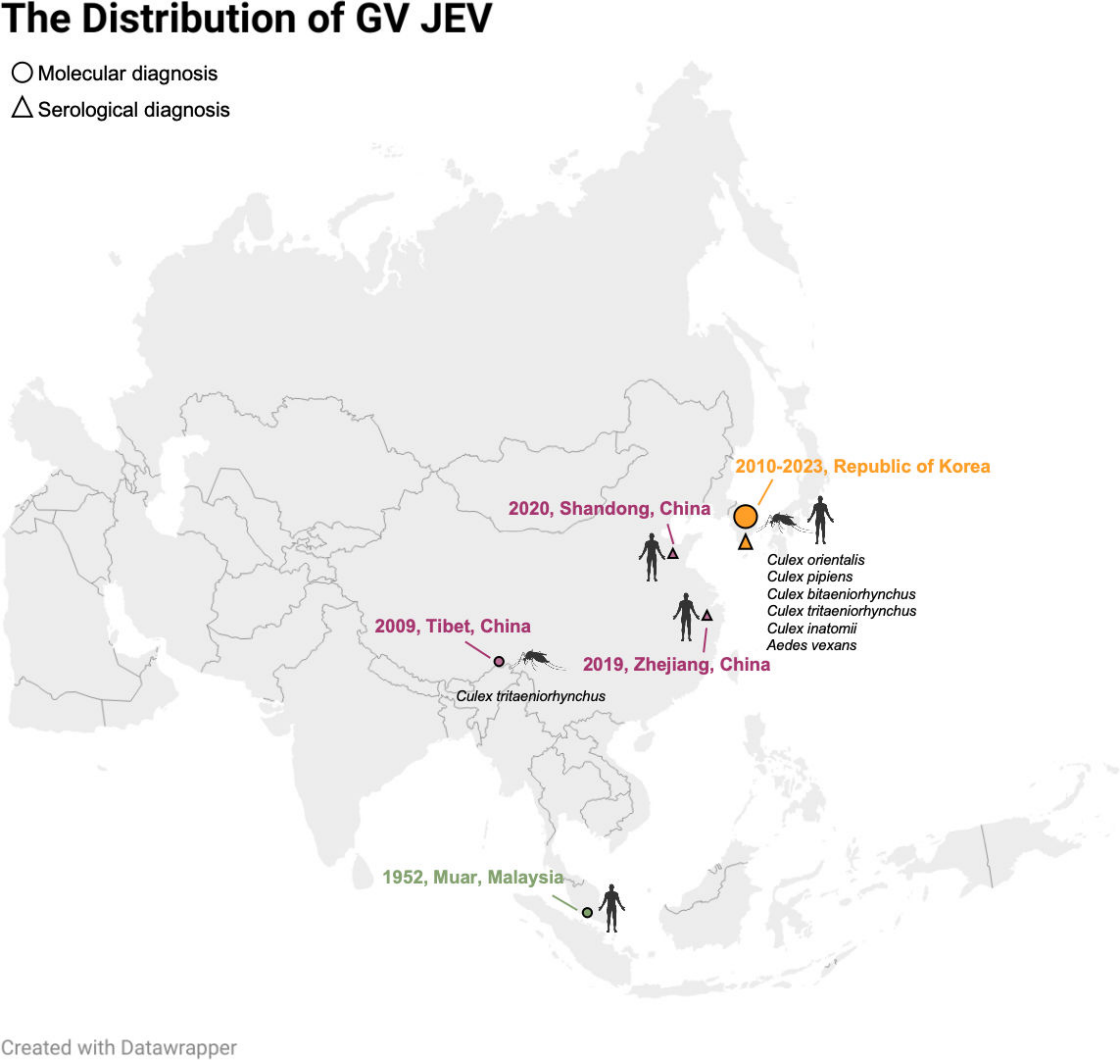
The distribution of GV JEV. In 1952, GV JEV was first isolated from a Japanese encephalitis patient in Muar, Malaysia (green marker). In 2009, it was re-detected in *Culex tritaeniorhynchus* in Nyingchi, Tibet, China (purple marker). Moreover, two human cases in 2019 and 2020 were identified by higher NAb titers against GV JEV than other genotypes. From 2010 to 2023, a total of 55 GV JEV strains were reported in the ROK, primarily from mosquito pools, with seven human cases, where four were also diagnosed by serological method (orange marker).

GV JEV can also cause human infection and death and has attracted increasing attention because it has been continuously detected in both mosquitoes and humans in the ROK, becoming almost the only genotype identified in mosquito pools in recent years (Fig. 1) (11–13). The genotype shift in the vector often signals potential changes in the predominant genotypes circulating in human populations. Moreover, current population immunity against GV JEV may be insufficient, and GV JEV has demonstrated higher lethality in mice compared to other genotypes (14). Therefore, this review summarizes the current understanding of GV JEV and discusses the protective efficacy of current vaccines against this genotype.

## EPIDEMIOLOGICAL CHARACTERISTICS OF GV JEV

### Patients

To date, 10 JE cases caused by GV JEV have been reported in Malaysia, China, and the ROK (Fig. 1). Four cases were confirmed by direct genomic detection of GV JEV (12, 15, 16), whereas the remaining six cases were identified through cross-neutralization testing of acute phase sera, in which neutralizing antibody (NAb) titers against GV JEV were higher than those against other genotypes (Fig. 1) (13, 17). The plaque reduction neutralization test assay for JEV is considered more sensitive than IgM-based diagnostics for JE (18). However, its reliability and applicability for JEV genotyping remain to be further explored, although several studies have suggested that NAb titers against GV JEV do not exceed those against other genotypes in the absence of GV infection (14, 19–21). In the future, this genotyping strategy should be evaluated for monitoring GV JEV in populations, since JEV can only be detected during a very limited period of viremia.

Among 10 cases, basic clinical information was available for four patients, ranging from children to adults, with both mild and severe manifestations observed in each age group. Notably, patients with a history of JE vaccination tended to present with milder symptoms, suggesting that the vaccine may still provide a certain degree of protection against the currently circulating GV JEV (15–17). In particular, disclosure of patient data in the ROK, which is the primary site of GV JEV detection, has been limited. This highlights the importance of information sharing for the prevention and control of GV JEV. Furthermore, current commonly used encephalitis pathogen detection kits do not routinely include JEV (22), which may have prevented timely recognition of its potential circulation. Moreover, in the context of widespread JE vaccination among both children and adults, infected individuals may only present with non-specific clinical symptoms (as reported in the ROK patient), which could lead clinicians to overlook the need for JEV testing, since the test is generally considered only in patients presenting with acute encephalitis (23). Therefore, in regions where JEV has historically circulated and has been detected, particularly in Southeast Asian countries, it has been imperative to strengthen JEV surveillance not only in patients with acute encephalitis but also in those with mild central nervous system infections. Once JEV infection is confirmed, timely genetic characterization is essential. Here, we emphasize the importance of updating genotyping methodologies, especially approaches capable of distinguishing all five JEV genotypes (24), which is critical in the context of multi-genotype JEV circulation.

### Vectors

**Fig 2:**
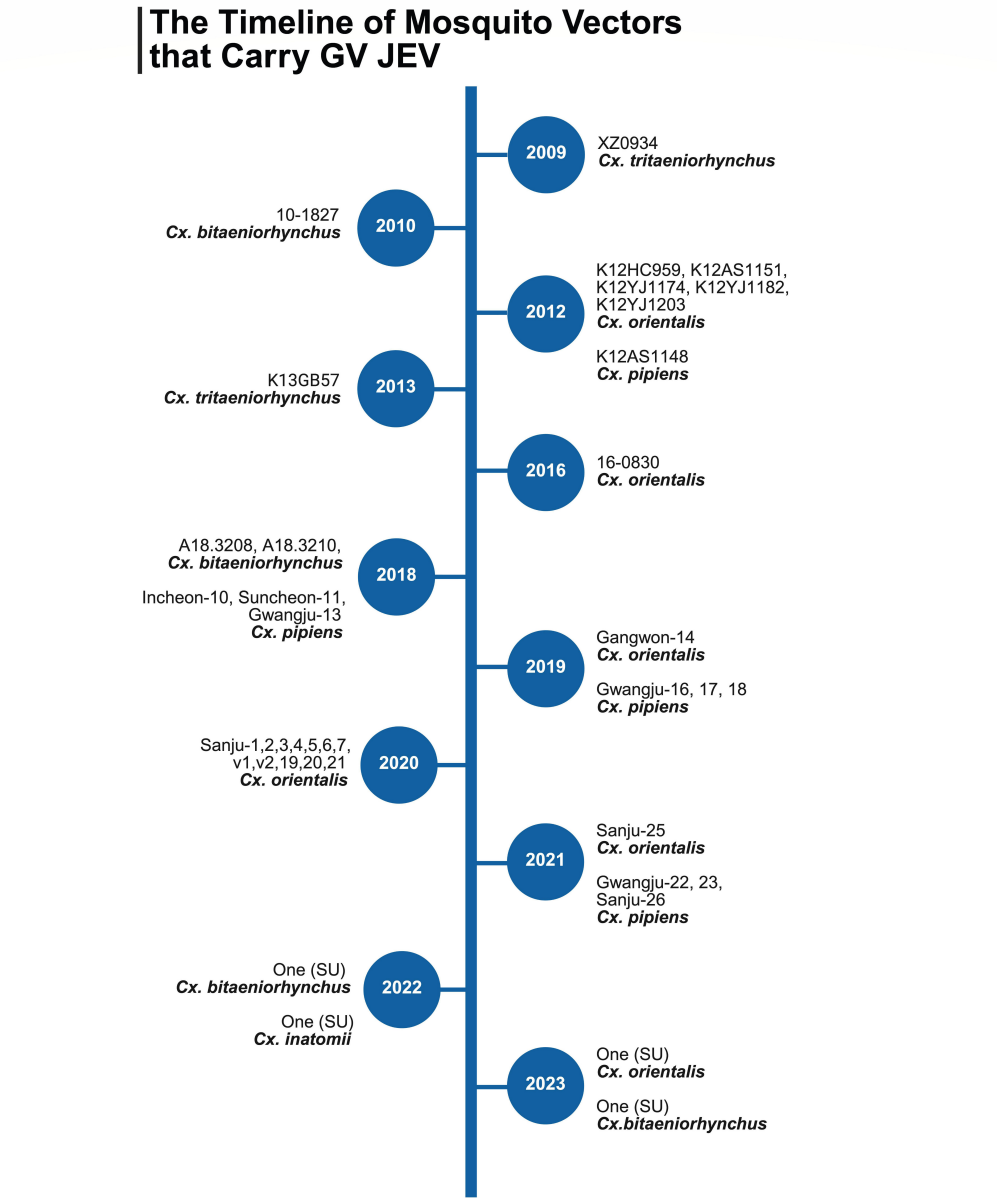
The timeline of mosquito vectors that carry GV JEV. This figure illustrates continuous detection of GV JEV from various mosquito species from 2009 to 2023 and indicates the species shift in GV JEV. SU indicates that sequence data are not publicly available but can be obtained from the authors upon request (28). SU, sequence unavailable.

JEV is maintained in an enzootic transmission cycle between mosquito vectors (primarily *Culex tritaeniorhynchus*) and vertebrate amplifying hosts (mainly pigs and wading birds). Recently, sheep have also been identified as amplification hosts for JEV (25). Humans and horses are considered dead-end hosts (26). The isolation of GV XZ0934 from *Culex (Cx.) tritaeniorhynchus* in China indicates its potential to establish human transmission cycle, as this species is the primary vector for JEV transmission to humans (Fig. 2) (27). However, during the period from 2010 to 2023 in the ROK, 48 GV JEV strains identified from mosquitoes were primarily associated with *Cx. orientalis* (50.0%, 24/48), *Cx. pipiens* (33.3%, 16/48), *Cx. bitaeniorhynchus* (10.4%, 5/48), *Cx. tritaeniorhynchus* (2.1%, 1/48)*, Cx. inatomii* (2.1%, 1/48), *and Aedes (Ae.) vexans* (2.1%, 1/48) (Fig. 2) (11, 28–30).

Prior to 2010, when GV JEV was found, over 70% of mosquito isolates carrying GI and GIII were identified as *Cx. tritaeniorhynchus*, whereas *Cx. orientalis* and *Cx. pipiens* together accounted for only about 2% (31–33). However, two recent longitudinal mosquito pool surveillance studies in the ROK showed that GV gradually became almost the sole detectable genotype from 2017 to 2023 (11, 28). These findings indicate a shift in both genotype prevalence and dominant mosquito vectors in the ROK. Moreover, *Cx. pipiens* (Italy, China), *Cx. bitaeniorhynchus* (India, Malaysia), and *Ae. vexans* (Taiwan) have been reported as JEV carriers in many areas (33). This suggests that regions outside the ROK may also have the potential to sustain GV JEV transmission. We therefore recommend increased collection of *Cx*. species, along with strengthened JEV surveillance and genotyping in these regions.

Pigs and birds are well-documented as amplifying hosts in the JEV transmission cycle (34). Birds may play a role in the genotype shift of JEV from GIII to GI (35). Moreover, sheep are susceptible to JEV and serve as a new amplifying host, with reported infection rates ranging from 40.6% to 57.1% (25). However, research on the role of these vectors in GV JEV transmission remains insufficient. Therefore, enhanced global surveillance and further investigation into the role of hosts will contribute to a better understanding of the transmission cycle of GV JEV.

## MOLECULAR BIOLOGICAL CHARACTERISTICS OF GV JEV

### Genome and sequence identity

JEV is a single-stranded positive-sense RNA virus with a genome of approximately 11 kb. Its genome consists of an open reading frame (ORF) and untranslated regions (UTRs) at both the 5′ and 3′ ends. The ORF encodes a polyprotein, which is cleaved by viral and host proteases into three structural proteins: capsid protein (C), precursor membrane protein (prM), and envelope protein (E), as well as seven nonstructural proteins: NS1, NS2A, NS2B, NS3, NS4A, NS4B, and NS5 (36).

From the perspective of genome length, GV JEV has a longer genome compared to the other four genotypes. The 5′-UTR length is consistent across all genotypes at 95 nucleotides (nt). However, the ORF of GV JEV is approximately 10,302 nt, which is 3 nt longer (AGC/AGT) than that of other genotypes. The 3 nt insertion is located in the NS4A region, resulting in the addition of a serine (178Ser) residue in the NS4A protein of GV JEV. Furthermore, the 3′-UTR of GV JEV is also relatively longer compared to other genotypes (37). NS4A and the 3′-UTR have previously been found to be associated with the replication and pathogenicity of JEV (38, 39). However, it remains unclear whether these differences are associated with GV JEV.

In addition, GV JEV exhibits differences in whole-genome nucleotide (78.9–79.3%) and ORF amino acid (90.6–91.2%) identities compared to the other four genotypes (37). Within GV JEV, a comparison of the available full-genome sequences of GV JEV, using the GV strain 16-0830 isolated in 2016 in the ROK (GenBank Number: MT568540) as the reference, revealed high sequence identity with two strains isolated in 2018 in the ROK, A18.3210 (MT568538) and A18.3208 (MT568539), with nucleotide identity of 99.2–99.4% and amino acid identity of 99.7–99.8%. However, the XZ0934 strain isolated in 2009 in China (JF915894) and the Muar strain (HM596272) showed nucleotide identities of 97.4% (amino acid: 99.5%) and 90.5% (98.2%), respectively, with the reference strain of 16-0830. These results indicated that the genetic divergence of GV JEV has increased over time and GV JEV is undergoing continuous evolution. We should note that the Muar strain was isolated from patient brain tissue inoculated into mouse brain (15), while the XZ0934 strain was obtained through inoculation of mosquitoes into BHK-21 (Baby Hamster Syrian Kidney) cells (27). The sequences of other strains were directly obtained through next-generation sequencing (40). Therefore, the methods of isolation may make contributions to variations in gene sequences.

### Phylogenetics

**Fig 3:**
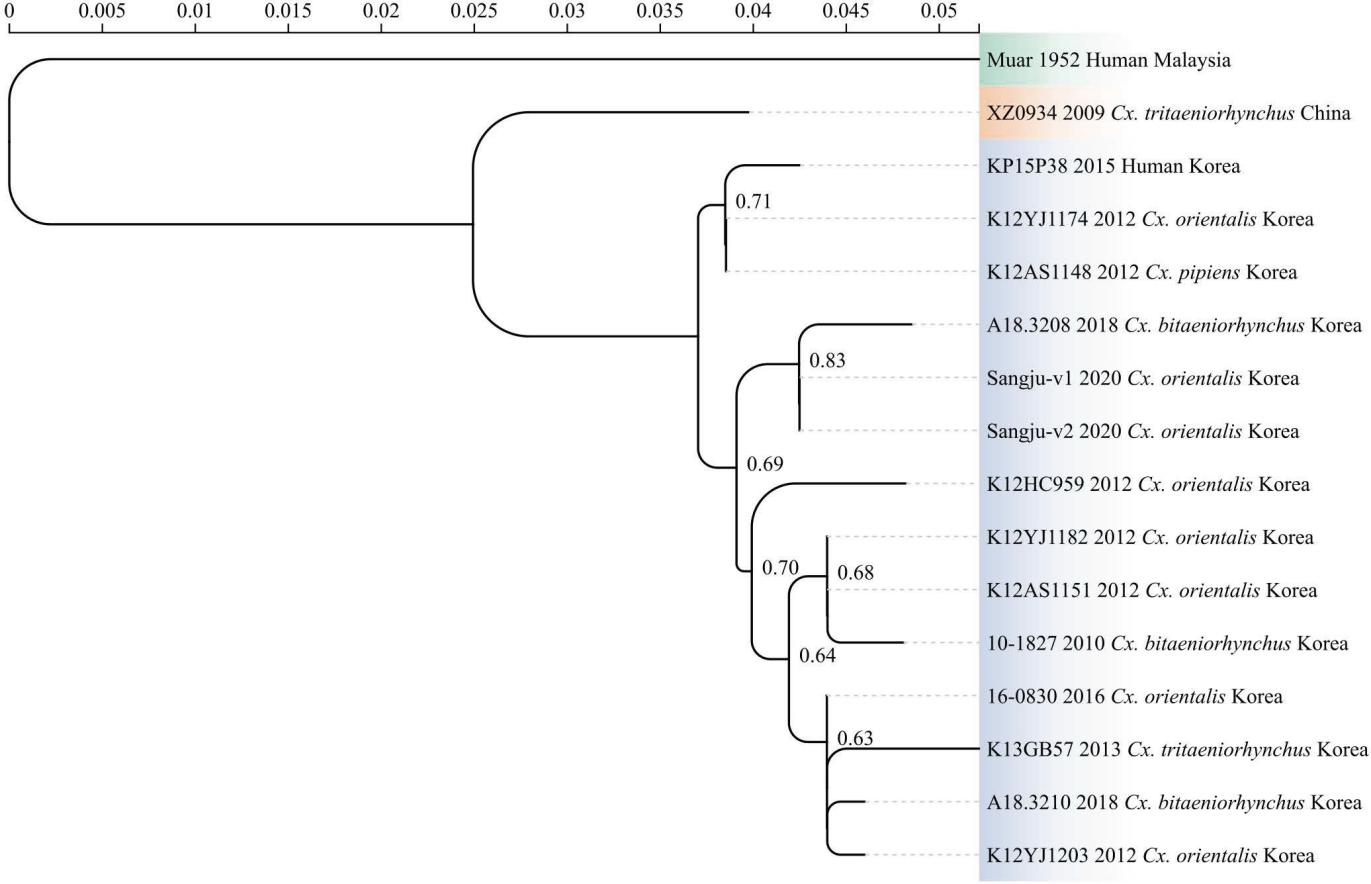
The phylogenetic analysis of GV JEV based on partial E protein sequence. The tree was constructed using the neighbor-joining method with the Kimura two-parameter model. The reliability of the branching was assessed with 1,000 bootstrap replications. Visualization of the tree was performed using Chiplot (44). The tree shows the evolutionary relationships among GV JEV strains isolated from humans and mosquitoes in Malaysia, China, and the ROK. Three major clades are evident: the Muar strain (1952, Malaysia), XZ0934 strain (2009, China), and ROK strains (2010–2020), reflecting accumulating genetic divergence within the GV genotype.

Phylogenetics serves as a commonly used tool for studying the origin and evolution of viruses based on the virus sequences (41). The origins and evolution analysis of JEV showed that the most recent common ancestor of JEV emerged approximately 3,255 years ago (95% highest posterior density: −978 to −6,125 years). Chronologically, the ancestral lineage gave rise to the five known genotypes of JEV in the following order: GV (−3,255 years), GIV (−1,653 years), GIII (−880 years), GII (−530 years), and GI (−155 years). This demonstrated that GV is the oldest genotype (42). Moreover, GV JEV has undergone evident evolutionary diversification within its lineage, forming three distinct clades: the Muar strain, the XZ0934 strain, and the ROK strains, which display progressively increasing evolutionary distances from the root of the phylogenetic tree. The ROK strains have further diverged into two sub-lineages, highlighting ongoing evolution within GV JEV (43). Overall, as the oldest genotype of JEV, GV JEV continues to undergo evolutionary divergence within its lineage (Fig. 3).

### Pathogenicity

Whether the sequence and evolutionary differences of GV JEV contributed to its pathogenicity remains an important question. *In vitro* experiments showed that GV JEV replicated at levels comparable to GI, GIII, and GIV strains in mosquito, avian, and other commonly used cell lines, but with reduced replication efficiency in neuronal cells. Consistently, GV JEV generally produced smaller plaques (21, 43, 45, 46) (Table 1). In contrast, *in vivo* studies demonstrated that GV JEV was associated with higher mortality rates than other genotypes (Table 1). Strain-specific differences have also been reported. One GV strain (43413), isolated from *Cx. orientalis*, displayed attenuated pathogenicity in both *in vitro* and *in vivo* studies. Other GV strains such as Muar and 43279 (from humans) and XZ0934 (from *Cx. tritaeniorhynchus*) did not show this attenuation. Given that approximately 50% of GV JEV isolates in the ROK were detected in *Cx. orientalis* and about 33% in *Cx. pipiens*, it remains unclear whether viruses carried by these species exhibit uniform reduced infectivity. Therefore, isolating and characterizing additional GV JEV strains from diverse mosquito vectors will be essential for clarifying their transmission dynamics and pathogenic properties.

**TABLE 1:** Comparison of GV JEV strains and other strains *in vitro* and *in vivo^a^*

GV strain	Reference strain	*In vitro* replication/plaque morphology	*In vivo* lethality	Effect on BBB	Reference
XZ0934	GIII RP-9	Replication: comparable in C6/36, DF-1, and SK-N-SH cells	C57BL/6 mice: comparable lethality BALB/c mice: higher lethality than GIII	Less efficient BBB penetration than RP-9	(45)
43279 43413 Muar	GIII Nakayama	Plaque size: Nakayama > Muar = 43279 > 43413	BALB/c mice: 43279 and Muar—higher mortality; 43413—non-lethal	43279 more permeable than 43413	(43)
Muar	GI Mie/41/2002 GIII Beijing-1	Plaque size: Muar = Beijing-1 < Mie/41/2002 Replication: comparable in Vero and BHK-21; slower in N18 cells	ddY mice: similar lethality to Beijing-1; both higher than Mie/41/2002		(46)
Muar	GI Mie/41/2002 GIV 19CxBa-83-Cv	Plaque size: Muar < 19CxBa-83-Cv < Mie/41/2002 Replication: comparable in Vero and IMR-32; slower in Neuro-2a cells	ddY mice: Muar > GIV 19CxBa-83-Cv > Mie/41/2002		(21)

^*a*^ DF-1: chicken fibroblast; SK-N-SH: human neuroblastoma; C6/36: mosquito-derived; BHK-21: hamster kidney; Vero: monkey kidney; IMR-32, Neuro-2a, N18: neuroblastoma cell lines.

In addition, blood–brain barrier (BBB) is a major factor influencing the neuropathogenesis of JEV (47). Although GV JEV exhibited stronger lethality and caused more severe brain pathology *in vivo* (43, 45), its infection efficiency in an *in vitro* BBB model was relatively low (45). This suggests that the pronounced neuroinvasive properties of GV JEV are not directly related to an increased ability to cross the BBB. Lee et al. (43) further analyzed BBB permeability *in vivo* and found it to be higher in strain 43279 compared to the less pathogenic strain 43413, although the study did not include other GV strains (42). The emergence of attenuated strains nonetheless provides an opportunity to investigate the mechanisms underlying the high virulence of GV JEV. Comparative sequence analyses combined with site-directed mutagenesis may help identify the viral determinants of pathogenicity.

### Viral determinants

Which viral determinants underlie the heightened pathogenicity of GV JEV in mice? To explore this, two chimeric viruses were generated by swapping the structural protein regions (C, PrM, and E) between GIII and GV JEV, yielding JEV S-GV/NS-GIII and JEV S-GIII/NS-GV. Infection experiments in mice showed that GV JEV and JEV S-GV/NS-GIII resulted in more severe neurological symptoms and higher mortality rates, suggesting that the structural protein region of GV JEV plays a key role in its neurovirulence (43). By employing the same strategy, E and PrM proteins were further identified as critical determinants (48). Moreover, site-directed mutagenesis found that the combination of substitutions in the E protein (N47K, L107F, H123R, E138K, and I176R) can reduce GV JEV’s neurotoxicity and neuroinvasiveness (49). Although these data suggest that the structural proteins, particularly the E protein, contribute to the virulence of GV JEV, comparison of strains 43279 and 43413 revealed eight amino acid differences across the ORF, with only two located in structural proteins and six in nonstructural proteins. Notably, none of the five substitutions previously implicated in virulence were found within the E protein (43). Therefore, further studies are needed to identify the specific determinants underlying GV JEV virulence.

## THE CONCERN ABOUT JE VACCINE AGAINST GV JEV

### The efficacy of current vaccines

Vaccination plays an important role in reducing JE incidence and current vaccines used in human populations, including inactivated and live-attenuated vaccines (LAVs), are all based on GIII strains (4). However, analyses of sequence, evolution, antigenicity, and pathogenicity have shown that GV JEV differs from GIII in several respects and may be more pathogenic (50, 51). Notably, GV has gradually replaced other genotypes and is now almost the only lineage detected in mosquitoes in certain regions (11). These differences highlight the potential risk of GV JEV circulation in humans and raise the question of whether current vaccines provide sufficient protection.

Compared to the prevaccination baseline, NAb seroprotection rates (SPRs, NAb ≥1:10) against GV JEV were enhanced after GIII vaccination in human populations (52). This indicates that the current vaccine can enhance resistance against GV JEV. However, LAV SA-14-14-2 and inactivated vaccines may have inadequate efficacy against GV JEV compared to other genotypes. Specifically, NAb SPRs and geometric mean titers are lower than those against GIII and GI (14, 52). Moreover, antibody levels against GIII JEV decline with age (53), and only high titers against GIII (>1:320) provide full protection against GV JEV (14). In recent years, most JE cases have occurred in adults, with individuals over 40 years of age accounting for up to 90% of cases (12, 54, 55). This suggests that NAb levels in adults may not be sufficient to protect against GV JEV.

NAb titers are sometimes undetectable, and the anamnestic NAb response can be rapidly activated upon re-exposure and provide protection (56). Nonetheless, in GIII-based vaccine-immunized mice, protection against GIII increases with higher vaccine doses. In contrast, although vaccinated mice displayed greater resistance to GV JEV than unvaccinated controls, this protection did not improve with increasing doses, where the protection level of NAb titers typically rises with increased vaccine doses (14, 57). This discrepancy suggests that factors beyond NAbs may be involved. Therefore, cellular immunity may also contribute to the protection, and we emphasize the importance of investigating whether T cells can provide effective immunity across different JEV genotypes, as such research would help clarify the mechanisms underlying these findings (58, 59). Furthermore, a vaccine breakthrough infection has been reported in the ROK, where GV JEV was detected in a previously vaccinated individual (16). Collectively, these findings indicate that current vaccines confer partial protection against GV JEV, but their protective efficacy requires further improvement.

### Essential evaluations before advancing GV JEV vaccine development

Given that no specific antiviral treatment is currently available for JEV, and considering the multifaceted risks posed by GV JEV, many studies have emphasized the urgent need for GV-based vaccine development (30, 52, 60). However, the development of a genotype-specific vaccine is an important but complex consideration. Several key aspects, therefore, warrant careful evaluation before advancing to clinical development. First, epidemiological data in human populations must be clarified, including the infection rate of GV JEV, vaccination history, age distribution, and disease severity of infected cases in the real world. These baseline data are essential for assessing whether vaccine development is necessary, and additional surveillance results are still needed to justify the rationale for developing a stand-alone vaccine against GV JEV. Second, protective immunity in existing populations needs to be thoroughly assessed, with particular attention to children, who are the main targets of routine immunization programs, and adults over 40 years of age, who often experience a decline in antibody levels with age. Such assessments should also address cross-reactive immunity against GV JEV and the presence of GV JEV-specific T cell responses. Third, continuous surveillance and molecular detection methods are indispensable. Obtaining complete viral sequences directly from patients is challenging, so more sensitive diagnostic and genotyping tools are needed, together with long-term monitoring of GV JEV population dynamics and genetic variation in mosquitoes and pigs, where increases in viral populations may precede large-scale human outbreaks and thus provide an early warning signal. Fourth, insights from animal models and immunological studies are crucial before clinical translation. Evidence indicates that GIII-based vaccines induce only partial protection against GV JEV (14). Moreover, GV-based vaccines can provide effective protection against GV JEV in mice, but they also display lower cross-protection against other genotypes (57, 61–63). Studies in mice, pigs, and non-human primates are required to validate the safety, immunogenicity, and protective efficacy of different vaccine strategies, including monovalent, multivalent, and recombinant approaches. Finally, international collaboration and public health strategies are essential. Although GV JEV has been predominantly detected in the ROK, past reports from China and Malaysia highlight the risk of cross-border transmission through mosquitoes and livestock. Addressing this threat will require coordinated regional and global One Health surveillance networks that share viral sequences, clinical data, and vector information, thereby enabling earlier detection of GV JEV spread and providing evidence for evaluating the global necessity of vaccine development (64, 65).

## CONCLUSION

**Fig 4:**
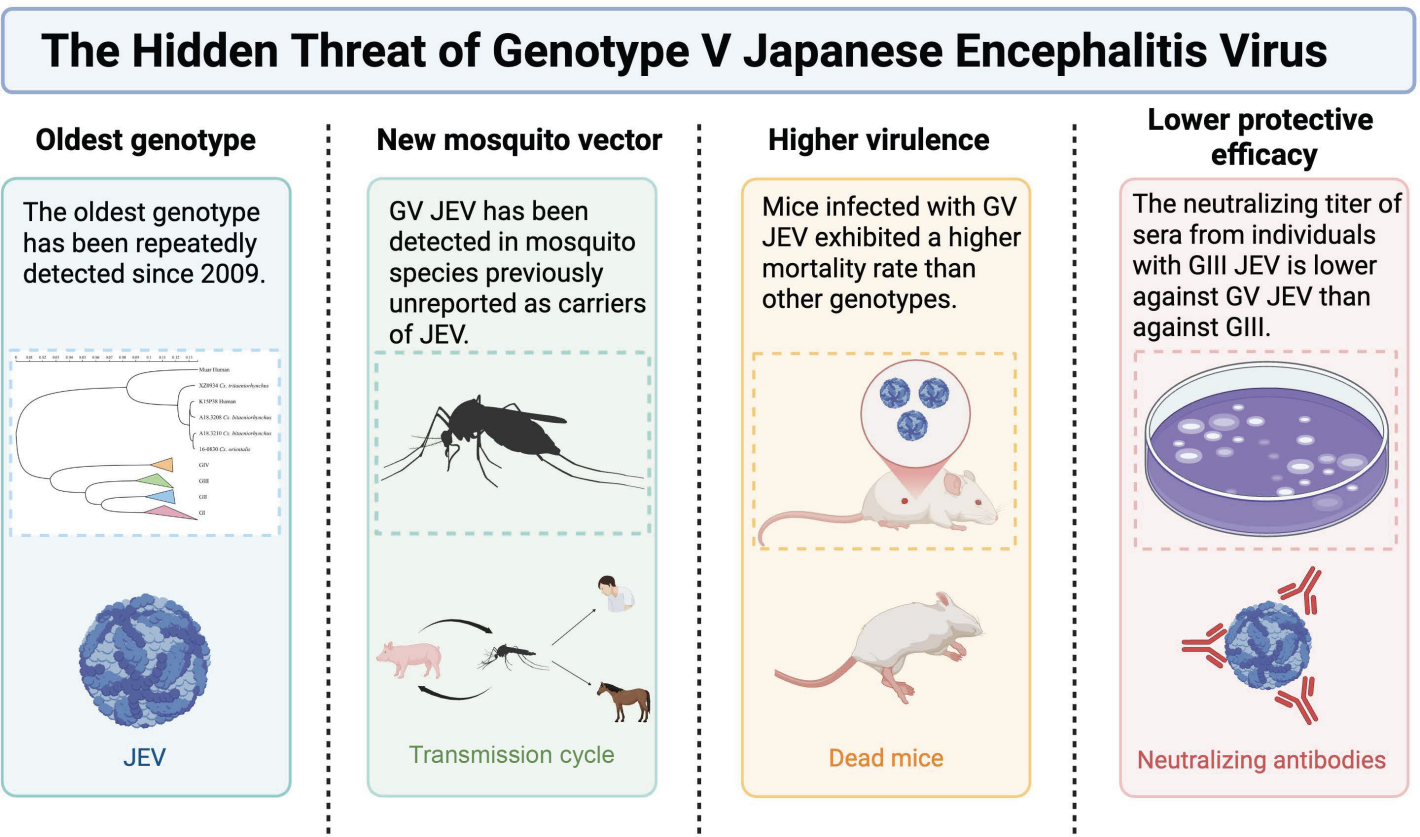
The hidden threat of genotype V Japanese encephalitis virus (GV JEV). This figure summarizes the key public health risks posed by GV JEV, including its increased pathogenicity in animal models, reduced vaccine efficacy, detection in novel mosquito vectors, and limited genomic surveillance. These factors highlight the urgent need for enhanced global monitoring, mechanistic studies, and the development of next-generation multivalent vaccines.

In conclusion, GV JEV exhibits differences from other genotypes in terms of genetic evolution, antigenicity, vector competence, and pathogenicity. Current vaccines provide partial but inadequate levels of protection. If GV JEV were to acquire increased transmission capacity, it could potentially drive a resurgence of JE (Fig. 4). However, both epidemiological and sequence data on GV JEV remain limited. We therefore call for the establishment of a global One Health surveillance network to complement national surveillance efforts by providing timely and comprehensive updates on genomic sequences, clinical data, and vector competence. Such coordinated efforts will facilitate investigations into the pathogenic mechanisms of GV JEV and inform the critical decision of whether the development of GV-based vaccines is warranted, thereby contributing to strategies aimed at mitigating this emerging public health threat.
